# Correction

**DOI:** 10.1111/cas.14382

**Published:** 2020-05-19

The Society would like to draw the reader's attention to an error in the following supplement:

“Abstracts of the 78th Annual Meeting of the Japanese Cancer Association; 2019 Sept 26‐28; Kyoto, Japan” as Cancer Science, Supplement 1, Vol. 110 (2019).

The following abstracts were not included in the supplement:

- P‐1295 to P‐1403 on 26 September 2019
- P‐2263 to P‐2387 on 27 September 2019

The supplement has been corrected and the missing abstracts are listed from page 765 onwards.

The Society apologizes for this error and any confusion it may have caused.

